# New Perspectives of Underlying Cardiomyopathy in Pediatric SMA Patients—An Age Matched Control Study

**DOI:** 10.3390/life15071091

**Published:** 2025-07-11

**Authors:** Georgiana Nicolae, Andrei Capitanescu, Madalina Cristina Leanca, Elena Neagu, Daniela Vasile, Cristina Filip, Eliza Cinteza, Amelia Aria, Bianka Maria Pavlov, Gabriela Uscoiu, Cristiana Ioana Raita, Andrada Mirea

**Affiliations:** 1 Faculty of Midwifery and Nursing, “Carol Davila” University of Medicine and Pharmacy, 020021, Bucharest, Romania; 2 “Dr. Nicolae Robanescu” Children’s Rehabilitation Center, 041408, Bucharest, Romaniaelenaneagu1097@gmail.com (E.N.);; 3 Department of Pediatric Nephrology, “M.S. Curie” Emergency Clinical Hospital for Children, 041451, Bucharest, Romania; 4 Faculty of Medicine, “Carol Davila” University of Medicine and Pharmacy, 020021, Bucharest, Romania; filipcristina06@yahoo.com (C.F.);; 5 Department of Pediatric Cardiology, “Marie Curie” Emergency Children’s Hospital, 041451, Bucharest, Romania; 6 Cardio-Thoracic Department, “Carol Davila” University of Medicine and Pharmacy, 021021, Bucharest, Romania; gabriela.uscoiu@live.com

**Keywords:** spinal muscular atrophy, cardiomyopathy, pediatric cardiology, echocardiography, cardiac abnormalities, neuromuscular disease, left ventricular mass, sinus tachycardia, electrocardiography

## Abstract

Spinal muscular atrophy (SMA) is a genetic neuromuscular disorder primarily affecting motor neurons. Emerging evidence suggests it also involves multiple organs, including potential cardiac manifestations. This study aimed to evaluate cardiac abnormalities in pediatric SMA patients compared to age-matched healthy controls, providing insight into underlying cardiomyopathy in this population. A total of 126 children were included in the study, with 63 SMA patients and 63 age-matched controls. We conducted clinical examinations, standard electrocardiography (ECG), and cardiac ultrasound (CUS) in all patients. Electrocardiographic analysis revealed a higher prevalence of sinus tachycardia in the SMA group and significantly deeper Q waves, indicating possible myocardial involvement. Echocardiographic findings demonstrated a significant reduction in left ventricular mass and left ventricular mass index in SMA patients compared to controls, despite normal systolic function. Statistical analysis confirmed that SMA diagnosis was an independent predictor of reduced myocardial mass, suggesting a distinct cardiac phenotype in SMA patients. This study provides new evidence of subclinical cardiac involvement in SMA, characterized by reduced myocardial mass, altered electrocardiographic parameters, and increased sinus tachycardia. These findings suggest a previously unrecognized form of cardiomyopathy in SMA that differs from cardiac manifestations typically seen in other neuromuscular disorders.

## 1. Introduction

Spinal muscular atrophy (SMA) is a severe genetic neuromuscular disorder resulting from homozygous mutations in the SMN1 gene. These mutations lead to progressive degeneration and loss of α-motor neurons in the spinal cord and brainstem. Consequently, affected individuals experience persistent and progressive muscle weakness [1,2].

The incidence of SMA is approximately 8 per 100,000 neonates, with a prevalence of approximately 1–2 per 100,000 individuals. Prior to the introduction of revolutionary disease-modifying treatments, which significantly improved disease progression, SMA was ranked as the second most common fatal autosomal recessive disorder after cystic fibrosis and was the most common genetic cause of infant mortality [2].

The SMN1 gene, located on chromosome 5, encodes SMN, a protein essential for motor neuron survival. The SMN2 gene closely resembles SMN1 and can exist in multiple copies, partially compensating for the absence of SMN1. Although the SMN2 gene produces significantly less SMN protein than the healthy SMN1 gene, additional copies of SMN2 are associated with less severe forms of SMA (types II–IV) [3].

The disorder shows a broad and heterogeneous spectrum. Numerous studies highlight its multiorgan involvement. Traditionally, SMAs are classified into five types based on the average age of onset and severity of symptoms:

1. SMA Type 0: The congenital and most severe form. It is characterized by early-onset muscular hypotonia in the neonatal period and is often accompanied by respiratory failure and feeding difficulties. The mortality rate is high during the first month of life, and most affected individuals do not survive beyond six months of age [4].
2. SMA Type 1 (Werdnig–Hoffmann disease or infantile-onset SMA): Onset typically occurs before 6 months of age. It is characterized by generalized hypotonia, developmental delay, and regression, alongside the absence of motor skill acquisition. Life expectancy is usually less than 2 years [5,6].
3. SMA Type 2 (chronic SMA): Typically manifests between 6 and 18 months of age. It is characterized by muscle weakness in the lower limbs, delayed motor skill acquisition, and the inability to stand independently. As muscle weakness progresses in childhood, affected individuals may require assistance while sitting. Life expectancy varies, but many live into their twenties or thirties [5].
4. SMA Type 3 (Kugelberg–Welander disease): Typically appears after 18 months of age. It presents with an unstable gait, frequent falls, and difficulty rising from a seated or lying position. All individuals with this condition can achieve independent walking. However, many patients require wheelchair assistance later in life. Life expectancy is generally normal [7].
5. SMA Type 4: Typically manifests after the age of 10 years. It is rare and is associated with mild to moderate muscle weakness, tremors, and mild respiratory difficulties. Normal life expectancy is observed [7].

The natural history of SMA varies significantly. It ranges from a high mortality rate (up to 95% in the first 18 months) in SMA type 1 to normal life expectancy in SMA types 2, 3, and 4 [8].

Until recently, treatment for SMA was primarily supportive. In 2017, two pivotal studies on novel treatments for SMA type 1 were published. They marked the advent of the first disease-modifying therapies and introduced a new era in SMA management. These treatments include Nusinersen (Spinraza) and Onasemnogene abeparvovec (Zolgensma). More recently, Risdiplam has further changed the therapeutic landscape, particularly in countries where it is available [9,10]. Nusinersen and Risdiplam modify mRNA splicing to increase the amount of functional SMN protein. Both require continuous use. Onasemnogene abeparvovec, a one-time somatic gene therapy, delivers healthy copies of the SMN1 gene into cells via a viral vector. These novel treatments have shown remarkable results and have changed the long-term prognosis of the disease by improving muscle movement, function, and survival [11].

## 2. Cardiac Manifestations of the Disease

SMA is now recognized as a multisystemic disease that affects more than just skeletal muscle. It also impacts organs such as the heart, kidneys, liver, pancreas, spleen, bones, connective tissues, and immune system. SMN is most highly expressed during embryonic development, explaining the impaired development of various tissues. Studies suggest that SMN deficiency significantly influences tissue degeneration with age [12].

Patients with SMA type 1, the most severe form, have a high mortality rate before 18 months of age. Thus, the introduction of novel treatments has facilitated the study of multiorgan involvement in this condition [13].

Several key studies have documented cardiac abnormalities in SMA, including congenital heart defects, valve disorders, cardiomyopathies, and electrocardiographic changes. These findings are most consistently observed in SMA types 0 and 1, although cardiac manifestations may occur in all four types of the disease [14].

Congenital cardiac malformations in the SMA typically present as mild abnormalities, such as interatrial and interventricular septal defects [15,16]. Rarely, more complex anomalies, such as hypoplastic left heart syndrome and atrioventricular septal defects, have been observed [17].

Electrocardiographic (ECG) abnormalities are prevalent in SMAs with baseline tremors and are often linked to peripheral muscle tremors in the SMA, which is a characteristic finding [18].

In addition to these baseline tremors, impulse initiation disorders are the most common cardiac rhythm abnormalities. Bradycardia, due to delayed electrical conduction, is reported most frequently and can lead to various types of atrioventricular and bundle branch blocks [19,20].

With respect to structural cardiac involvement, studies on mouse models have shown reductions in myocardial wall thickness and decreased heart size, with myocardial fibrosis present [21,22].

These findings suggest a direct impact of SMN deficiency on cardiac tissue. Structural cardiac malformations occur more often in patients with severe SMA (e.g., type 1), whereas arrhythmias appear more frequently in milder forms (types 2 and 3). This underscores the heterogeneous nature of the SMA and its manifestations across organ systems [14].

The exact mechanisms underlying cardiac abnormalities in the SMA are not fully understood. A study by Lei Sheng et al. (2017) explored these mechanisms, highlighting the role of survivin, a crucial protein for cardiac development, in abnormal cell cycle progression and apoptosis in cardiomyocytes [23]. Another study revealed significantly increased mRNA expression of genes typically upregulated in heart failure heart tissue from an autopsy series of SMA patients [12].

The peripheral role of SMN proteins in each organ and their contributions to embryological development, tissue function, and survival remain insufficiently understood.

## 3. Materials and Methods

We performed a comparative study between 63 SMA patients and 63 age-matched healthy controls, with no structural cardiac disease or systemic condition. Our primary goal was to evaluate the prevalence and range of cardiac abnormalities in SMA patients via advanced diagnostic tools. The SMA cohort included 63 pediatric patients who were diagnosed with SMA via genetic testing between June 2023 and July 2024. This group comprised 37 patients with SMA type 1, 21 with SMA type 2, and 5 with SMA type 3. Genetic testing also determined the number of SMN2 copies. This study exclusively enrolled a DMT-treated SMA cohort receiving ongoing disease-modifying therapy (nusinersen, risdiplam, or onasemnogene abeparvovec), contrasting with age-matched healthy controls. This therapeutic variable distinguishes our cohort from natural history studies of untreated SMA populations. The control group included 63 healthy pediatric individuals matched by age, as recommended by general practitioners for mild systolic murmurs or chest pain, who did not have any structural cardiac disease or systemic condition. Among the SMA patients, 37 had SMA type 1, 21 had SMA type 2, and 5 had SMA type 3. Of those tested, 24 had two copies of SMN2, 17 had three copies, and 3 had four copies. See the table below (Table 1).

We conducted clinical examinations, standard electrocardiography (ECG), and cardiac ultrasound (CUS) in all patients. Those with ECG-detected arrhythmias underwent 24-h Holter monitoring. The ECG parameters assessed included heart rhythm, heart rate, QRS axis, PR interval, QRS complex, corrected QT interval, baseline tremor, Q-wave amplitude, and conduction disturbances. All participants underwent comprehensive echocardiographic exams via the EPIQ7 ultrasound system (Philips Medical Systems, Best, Netherlands). Standard parasternal long-axis (PLAX), parasternal short-axis (PSAX), apical four-chamber (AP4), two-chamber (AP2), subcostal, and suprasternal views were obtained with a frame rate of 60–90 frames per second.

To assess the left ventricular myocardial mass (LVM), we used the following formula:

$$\mathrm{LVM}\;=\;0.8\times \left\{1.04\times \left[\left({\left(\mathrm{LVEDD}+\mathrm{IVSd}+\mathrm{PWd}\right)}^{3}-{\left(\mathrm{LVEDD}\right)}^{3}\right)\right]\right\}+0.6\;(\mathrm{units}:\;g)$$

Legend: LVM = left ventricular mass; LVEDD = left ventricular end-diastolic diameter; IVSd = interventricular septal thickness in diastole; PWd = posterior wall thickness in diastole.

Since patients with SMA exhibit staturo-ponderal hypotrophy, for an accurate assessment of left ventricular mass, we considered it necessary to index the LVM to the body surface area, calculating the LVMI using the following formula:

$$\mathrm{LVMI}=\frac{\mathrm{LVM}}{\mathrm{BSA}}\;(\mathrm{units}:\;{g/m}^{2})$$

Legend: LVMI = left ventricular mass index; BSA = body surface area.

Statistical analyses were performed via Analyze IT 6.15 (Microsoft Office Excel add-on, Leeds, UK) and IBM SPSS Statistics for Windows, Version 26 (IBM Corp, Armonk, NY, USA). Continuous data is presented as medians with interquartile ranges, and categorical data are presented as numbers and percentages. We used non-parametric tests to compare quantitative parameters, and chi-square tests were employed for qualitative data. A *p* value less than 0.05 was considered statistically significant.

## 4. Results

A total of 126 patients were included, 42.1% of whom were male. Ages ranged from 1 month to 16 years (Table 2).

### 4.1. Electrocardiographic (ECG) Findings

**Figure 1:**
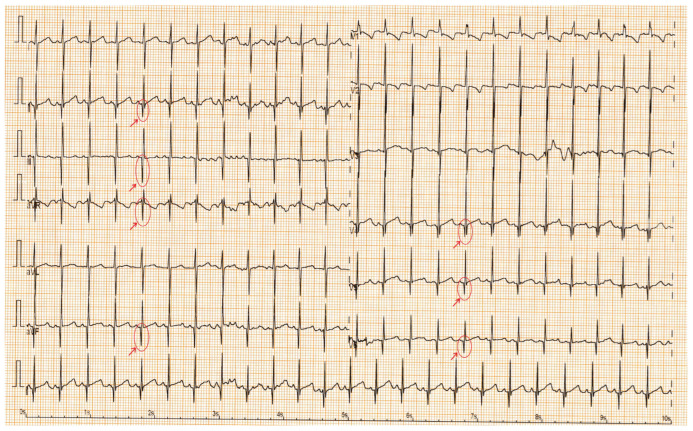
ECG with tremor, sinus tachycardia; red arrows highlight deep Q waves in DII, DIII, aVF, aVR, V4–V6.

The ECGs show mild changes, with no significant abnormalities. A sinus rhythm was observed in all the children. The PR interval, QRS duration, QRS axis, and QTc interval were within normal limits for both groups. Sinus tachycardia was found in 23.8% (15 patients) of the SMA group and in 1.6% (1 patient) of the control group. Sinus bradycardia was noted in 3.2% (2 patients) of the SMA cohort. The median heart rate percentile was 50 in the control group and 75 in the SMA group (*p* < 0.01). The Q-wave amplitude was significantly greater in the SMA group than in the control group, with a median amplitude of 4 mm and 3 mm, respectively (*p* = 0.01) (Figure 1). The higher prevalence of RBBB patterns in controls (57.14%) compared to SMA patients (28.57%) likely reflects benign, age-related ECG variants common in healthy children, rather than disease-specific conduction abnormalities (Table 3).

### 4.2. Echocardiographic Findings

**Figure 2:**
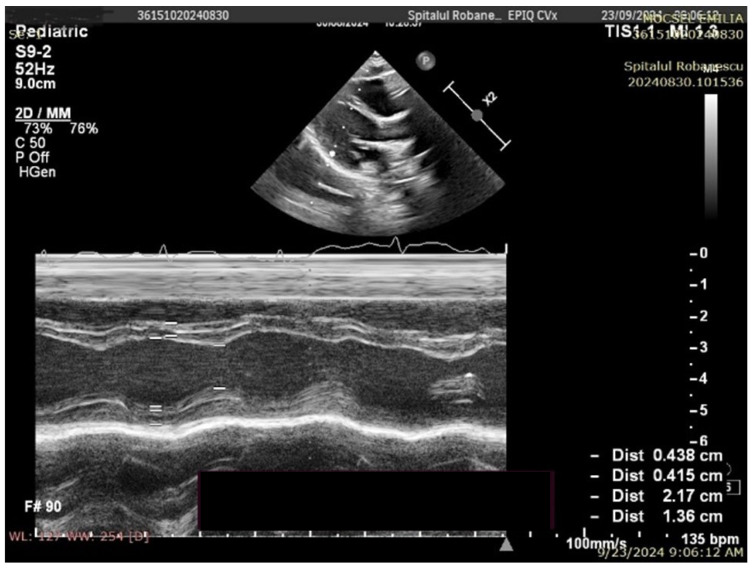
Transthoracic echocardiography in an SMA type I patient (8 months old, 7.4 kg)—M-mode measurements (LVM = 14.24 g, *p* = 0.05%, Z score −3.27).

**Figure 3:**
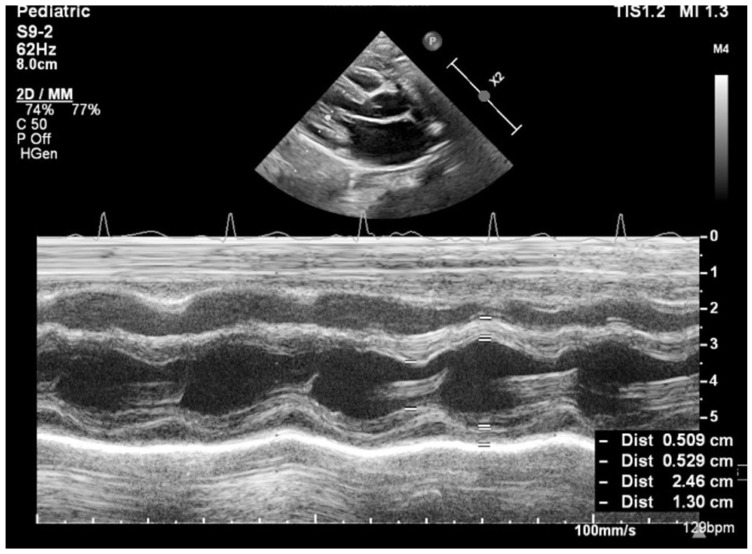
Transthoracic echocardiography in a control group patient (8 months old, 8 kg)—M-mode measurements (LVM = 21.69 g, *p* = 38.97%, Z score −0.29).

No complex congenital cardiac malformations were detected in the SMA cohort. Owing to thoracic deformities (common in the SMA), the left ventricular ejection fraction (EF) cannot be accurately measured via the Simpson method or strain rate studies; instead, fractional shortening (FS) was used to assess systolic function. All the children had normal systolic functions. Mild septal defects, such as patent foramen ovale (PFO) or small atrial septal defects (ASD), were observed. The left ventricular mass (LVM) percentile and left ventricular mass index (LVMI) significantly differed between the SMA and control groups. The median LVM percentile was 1.98% in the SMA group compared with 13.57% in the control group (*p* < 0.01). Similarly, the median LVMI was 50.54 in the SMA cohort and 56.27 in the control cohort (*p* < 0.01) (Figure 2 and Figure 3, Table 3).

### 4.3. Binomial Regression

The LVMI is closely related to age and sex. To test our findings, we divided our cohort into 2 classes based on the median LVMI. We performed binomial multiple regression, choosing this class as the dependent variable. The presence of SMA and age were retained in the last model, with odds ratios of 0.227 and 1.027, respectively, with the CI excluding 1 (Table 4).

## 5. Discussion

To our knowledge, this is the largest study involving pediatric SMA patients, with the mention that all patients with SMA received disease-modifying treatment at the time the study was conducted. It includes the most extensive cohort of children with SMA type 1, directly compared with a control group, and reveals significant differences in cardiac parameters between the two populations. Additionally, our study provides valuable insights into the cardiac manifestations of this neuromuscular disorder.

The universal administration of disease-modifying agents to our SMA cohort establishes a fundamental pathophysiological distinction from untreated SMA populations described in medical literature. Consequently, observed cardiac profiles represent a treatment-influenced disease state, potentially obscuring or modifying innate SMA-associated cardiac manifestations. This therapeutic confounder must be considered when extrapolating findings to untreated SMA populations.

From an echocardiographic perspective, significant cardiac malformations were not identified; only mild anomalies, such as atrial septal defects, patent foramen ovale, and patent ductus arteriosus, were found. These anomalies paralleled those observed in the controls. The most striking result was the reduced left ventricular mass (LVM). The median LV mass percentile was substantially lower in the SMA group than in the control group, and the difference was statistically significant. This parameter correlated with disease severity and lower body weight in SMA patients; those with SMA type 1 had the lowest myocardial mass indices in the present study.

With respect to the ECG findings, no severe rhythm or conduction disturbances were noted. Only one case of isolated supraventricular extrasystole was observed. However, sinus tachycardia occurred more frequently in the SMA group. Another significant observation was the presence of more pronounced Q waves in the SMA cohort, extending to leads beyond those typically considered normal.

The combination of a reduced left ventricular mass, sinus tachycardia, and pathological Q waves points to a unique form of cardiac involvement in SMA. These findings indicate the existence of an underlying cardiomyopathy in patients with SMA, which is distinct from the cardiomyopathies typically observed in other neuromuscular disorders.

Most data on cardiac changes in SMA comes from animal models, which have demonstrated that cardiomyocyte impairment occurs through decreased proliferation and increased apoptosis. This results in fewer, smaller cardiomyocytes and greater collagen deposition relative to healthy hearts [22]. In human models, only isolated case reports have described SMAs associated with cardiac malformations, arrhythmias, or conduction disorders [14].

We reviewed the published literature on cardiovascular complications associated with neuromuscular disorders. Our conclusion is that the cardiac phenotype observed in patients with SMA exhibits distinct characteristics not previously described in the context of neuromuscular disease-related cardiac involvement. Evidence from multiple animal model studies supports this finding.

Duchenne and Becker muscular dystrophies are predominantly linked to dilated cardiomyopathy (DCM), whereas Friedreich’s ataxia is associated with hypertrophic cardiomyopathy (HCM) and various arrhythmias [24,25].

Similarly, mitochondrial cardiomyopathies can present as either DCM or HCM [26]. The cardiac features identified in SMA patients more closely resemble prematurity-related cardiomyopathy rather than typical cardiomyopathic patterns commonly reported in neuromuscular disorders [27].

Prematurity-related cardiomyopathy has been known for more than two decades; at birth, fetal cardiac and arterial proliferation and development abruptly decelerate. This disruption affects the normal process of cardiomyocyte differentiation in preparation for postnatal life. Animal studies have demonstrated that hearts in preterm models are smaller with a reduced number of binucleated cardiomyocytes following premature birth. Studies on ovine models have shown a sevenfold increase in diffuse collagen deposition compared with that in term hearts. Additionally, murine models have revealed shortened, disorganized myofibrils that fail to align properly within the myocardium of preterm subjects [28].

In a study by Mohlkert et al. that utilized echocardiography, the left ventricle (LV) was significantly smaller in size, with altered function, in children and adolescents born preterm. These findings indicate a fourfold increased risk of heart failure in those born from 28–31 weeks of gestation. The risk for individuals born before 28 weeks is increased by 17-fold [29].

Additionally, in preterm infants, a smaller heart with thinner walls and altered geometry results in reduced blood flow through the superior mesenteric artery. This affects intestinal absorption and weight gain [30].

In studies on animal models of spinal muscular atrophy (SMA), murine models have demonstrated increased collagen deposition, reduced heart size, and a diminished response to dobutamine stress. All these parameters indicate impaired contractile reserve. These studies revealed a thin and branched left ventricular wall in severe embryonic SMA hearts compared with a thick and well-developed left ventricular wall in wild-type embryonic hearts [22].

A recent study involving patients with spinal muscular atrophy (SMA) types 2 and 3, who had not received disease-modifying therapy, evaluated cardiac function and identified biventricular systolic and diastolic dysfunction, as indicated by reduced longitudinal strain [31].

Cardiac alterations encountered in SMA patients include a thinner-walled left ventricle, more frequent sinus tachycardia, and the presence of Q waves suggestive of subclinical myocardial involvement. These could be among the contributing factors to heart failure in the case of severe respiratory infections. These factors might also contribute to malnutrition in these patients.

This study underscores the importance of cardiac monitoring in SMA patients. This highlights the need for further research, including advanced imaging techniques and molecular studies, to fully understand the extent of cardiac involvement in the SMA and to develop standardized evaluation protocols.

## 6. Limitations

We admit, as a limitation of our study, the inability to accurately measure the left ventricular ejection fraction (EF) via the Simpson method or strain rate studies because thoracic deformities are commonly present in SMA patients. As a result, fractional shortening (FS) was employed to assess systolic function instead. NT-proBNP assessment was omitted because of resource limitations, despite its potential to indicate increased wall stress or subclinical heart failure.

All SMA patients in our cohort were receiving disease-modifying therapies (nusinersen, onasemnogene abeparvovec, or risdiplam) at the time of evaluation. This is a significant distinction from most historical studies of cardiac involvement in SMA, which largely describe untreated populations.

The absence of an untreated SMA comparator cohort limits the ability to definitively ascribe observed cardiac manifestations exclusively to SMA pathology, as opposed to potential effects mediated by disease-modifying therapies (DMTs).

## 7. Conclusions

Diagnosing heart failure in patients with SMA can be challenging because their limited physical activity often conceals early signs of cardiac compromise. The fatigue observed in these patients is usually attributed to neuromuscular weakness. Our data demonstrates that SMA patients exhibit cardiac abnormalities on both ECG and CUS. Specifically, there was a significantly reduced left ventricular mass. This is accompanied by sinus tachycardia and pathological Q waves. These findings suggest the possibility of underlying cardiomyopathy, which appears to be distinct from that observed in neuromuscular disorders such as Duchenne or Becker muscular dystrophy. In such disorders, the heart typically shows dilatation and reduced contractility. Instead, SMA-related cardiomyopathy more closely resembles features found in premature infants. In this context, further research would be highly beneficial. Advanced imaging techniques, such as cardiac magnetic resonance imaging (MRI) and dobutamine stress tests, could enhance our understanding. Molecular studies of cardiomyocytes could also contribute valuable insights. Such investigations could help establish standardized protocols that are essential for the evaluation and treatment of cardiac complications in SMA patients.

## Figures and Tables

**Table 1:** SMA patient characteristics.

AMS	
Type I	37
Type II	21
Type III	5
**Treatment**	
Nusinersen	51
Onasemnogene abeparvovec	6
Risdiplam	6
**SMN gene copies**	
2 copies	34
3 copies	25
4 copies	4

**Table 2:** Cohort characteristics.

Parameter	Overall (*n* = 126)
Male gender (%)	53 (42.1)
Age (months)	46 [17.9; 103.2]
Age (years)	3.5 [1; 8]
Weight (kg)	14 [10; 25]
Weight (p%)	37 [9; 70]
Height (cm)	99 [82.9; 128]
BSA	0.62 [0.48; 0.89]
ECG	
Tremor present	32 (25.4%)
Rhythm	
Sinus	108 (85.7%)
Sinus tachycardia	16 (12.7%)
Sinus bradycardia	2 (1.6%)
QRS axis (degrees)	70 [60; 90]
BPM	114 [95; 130]
BPM percentile	75 [50; 90]
PR Length (ms)	120 [100; 140]
Q max amp (mm)	3 [2; 5]
RBBB (%)	54 (43.5)
QRS length (ms)	80 [67.8; 80]
QTc length (ms)	420 [400; 433]
Cardiac ultrasound	
LVM (g)	33.9 [21.44; 54.09]
LVM percentile	6.49 [1.24; 19.49]
LVMI	53 [46.36; 61.46]

**Table 3:** Cohort characteristic univariate analysis.

Parameter	SMA (*n* = 63)	Control (*n* = 63)	*p* Value
Male gender (%)	25 (39.7)	28 (44.4)	0.58
Age (months)	44 [20.2; 97.8]	48 [17; 108.3]	0.95
Weight (kg)	12.5 [9.58; 22]	16.2 [10.17; 29.67]	0.13
Weight (p%)	11 [1; 52]	57 [30; 77]	<0.01
Height (cm)	98 [82.2; 121.8]	102 [84; 139.3]	0.44
BSA	0.59 [0.45; 0.88]	0.67 [0.5; 1.06]	0.2
ECG			
Tremor present	31 (49.2%)	1 (1.6%)	<0.01
Rhythm			<0.01
Sinus	46 (73%)	62 (98.4%)	
Sinus tachycardia	15 (23.8%)	1 (1.6%)	
Sinus bradycardia	2 (3.2%)	0 (0%)	
QRS axis (degrees)	73 [60; 89.2]	70 [60; 90]	0.82
BPM	120 [100; 140]	103 [86.2; 124.8]	<0.01
BPM percentile	78.5 [50; 90]	50 [25; 73.7]	<0.01
PR Length (ms)	120 [100; 130]	120 [100; 140]	0.23
Q max amp (mm)	4 [2.02; 5]	3 [2; 3]	0.01
RBBB	18 (28.57%)	36 (57.14%)	<0.01
QRS length (ms)	80 [70; 80]	80 [60; 80]	0.89
QTc length (ms)	425 [406; 439]	413 [395; 426]	0.02
Cardiac ultrasound			
LVM (g)	32.76 [19.45; 47.88]	36.23 [25.26; 62.82]	0.05
LVM percentile	1.98 [0.32; 9.94]	13.57 [5.48; 25.08]	<0.01
LVMI	50.54 [43.04; 55.68]	56.27 [47.46; 64.82]	<0.01

**Table 4:** Binomial regression.

		Sig.	Exp(B)	95% CI for Exp(B)	
				**Lower**	**Upper**
Final Step	Age (months)	0.000	1.027	1.016	1.038
	SMA	0.001	0.227	0.094	0.552

Variables entered on first step: SMA, Age (months), weight (percentile), BPM (percentile), Rhythm, tremor, QTc msec, Qmax amplitude.

## Data Availability

The datasets generated and analyzed during the current study are not publicly available but are available from the corresponding author upon reasonable request.

## Abbreviations

The following abbreviations are used in this manuscript:

AP2	apical two chamber view
AP4	apical four chamber
ASD	atrial septal defect
BPM	beats per minute
BSA	body surface area
CI	Confidence interval
CUS	cardiac ultrasound
DCM	dilated cardiomyopathy
DMT	disease modifying therapy
ECG	Electrocardiography
EF	left ventricular ejection fraction
FS	fractional shortening
HCM	hypertrophic cardiomyopathy
IVSd	interventricular septal thickness in diastole
LV	left ventricle
LVEDD	left ventricular end-diastolic diameter
LVM	left ventricular myocardial mass
LVMI	left ventricular myocardial mass index
MRI	magnetic resonance imaging
mRNA	messenger ribonucleic acid
NT-proBNP	N-terminal pro b-type natriuretic peptide
PFO	patent foramen ovale
PLAX	parasternal long axis view
PSAX	parasternal short axis view
PWd	posterior wall thickness in diastole
RBBB	right bundle branch block
SMA	Spinal muscular atrophy
SMN1	Survival Motor Neuron 1
